# Oxidative Formation and Removal of Complexed Mn(III) by *Pseudomonas* Species

**DOI:** 10.3389/fmicb.2018.00560

**Published:** 2018-04-12

**Authors:** Mitchell H. Wright, Kati Geszvain, Véronique E. Oldham, George W. Luther, Bradley M. Tebo

**Affiliations:** ^1^Division of Environmental and Biomolecular Systems, Oregon Health & Science University, Portland, OR, United States; ^2^Department of Biology, Lynchburg College, Lynchburg, VA, United States; ^3^School of Marine Science and Policy, University of Delaware, Lewes, DE, United States; ^4^Department of Marine Chemistry and Geochemistry, Woods Hole Oceanographic Institution, Woods Hole, MA, United States

**Keywords:** Manganese(III), Mn(III)-DFOB, Mn(III)-citrate, Mn(III)-L, *Pseudomonas*, bacterial manganese oxidation

## Abstract

The observation of significant concentrations of soluble Mn(III) complexes in oxic, suboxic, and some anoxic waters has triggered a re-evaluation of the previous Mn paradigm which focused on the cycling between soluble Mn(II) and insoluble Mn(III,IV) species as operationally defined by filtration. Though Mn(II) oxidation in aquatic environments is primarily bacterially-mediated, little is known about the effect of Mn(III)-binding ligands on Mn(II) oxidation nor on the formation and removal of Mn(III). *Pseudomonas putida* GB-1 is one of the most extensively investigated of all Mn(II) oxidizing bacteria, encoding genes for three Mn oxidases (McoA, MnxG, and MopA). *P. putida* GB-1 and associated Mn oxidase mutants were tested alongside environmental isolates *Pseudomonas hunanensis* GSL-007 and *Pseudomonas* sp. GSL-010 for their ability to both directly oxidize weakly and strongly bound Mn(III), and to form these complexes through the oxidation of Mn(II). Using Mn(III)-citrate (weak complex) and Mn(III)-DFOB (strong complex), it was observed that *P. putida* GB-1, *P. hunanensis* GSL-007 and *Pseudomonas* sp. GSL-010 and mutants expressing only MnxG and McoA were able to directly oxidize both species at varying levels; however, no oxidation was detected in cultures of a *P. putida* mutant expressing only MopA. During cultivation in the presence of Mn(II) and citrate or DFOB, *P. putida* GB-1, *P. hunanensis* GSL-007 and *Pseudomonas* sp. GSL-010 formed Mn(III) complexes transiently as an intermediate before forming Mn(III/IV) oxides with the overall rates and extents of Mn(III,IV) oxide formation being greater for Mn(III)-citrate than for Mn(III)-DFOB. These data highlight the role of bacteria in the oxidative portion of the Mn cycle and suggest that the oxidation of strong Mn(III) complexes can occur through enzymatic mechanisms involving multicopper oxidases. The results support the observations from field studies and further emphasize the complexity of the geochemical cycling of manganese.

## Introduction

The geochemical cycling of manganese (Mn) in aquatic and terrestrial systems is largely governed by microbial oxidative and reductive processes, involving three oxidation states of Mn (II, III, and IV). Under anoxic conditions, bacterial reduction of insoluble Mn(III,IV) oxide minerals to soluble Mn(II) can occur as an energy generating process, involving the consumption of organic carbon coupled to oxides as terminal electron acceptors (Myers and Nealson, 1988). Conversely, in the presence of oxygen, bacteria are capable of oxidizing Mn(II) to Mn(III,IV) oxide though the purpose for this process is less understood (Tebo et al., 2004, 2005; Wright et al., 2016). While the processes that govern Mn(II) oxidation and solid Mn(III,IV) reduction have been well-studied, it has recently been observed that soluble Mn(III) bound to organic complexing ligands (Mn(III)-L) can dominate marine systems, comprising up to 100% of the total dissolved manganese (Oldham et al., 2017b). These complexes are defined as weak [Mn(III)-L_(weak)_] or strong [Mn(III)-L_(strong)_] as per their relative conditional stability constants (Oldham et al., 2015). This increased understanding of Mn(III) speciation has fundamentally changed the way we look at the role of environmental manganese. Mn(III)-L is capable of either donating or accepting electrons which subsequently significantly influences the redox chemistry of the surrounding environment (Luther et al., 2015). While research has highlighted the presence and significance of Mn(III)-L in these systems from a geochemical standpoint, little is known about the biological processes contributing to Mn(III)-L accumulation or removal.

In aquatic systems, the formation of Mn(III)-L is largely dependent on the surrounding redox conditions which are subject to change, not only through microbial influence, but also through various abiotic chemical processes. Environments containing high levels of hydrogen sulfide (H_2_S) can alter the relative prevalence of Mn(III,IV) oxide through redox transformations, producing reduced manganese and elemental sulfur (Herszage and Afonso, 2003). In iron rich systems, Fe^2+^ reduces Mn(III,IV) oxide at rates that can often outpace bacterial reduction (Siebecker et al., 2015). Furthermore, reactive superoxide (${\text{O}}_{2}^{-}$) can drive the non-enzymatic oxidation of Mn(II) and is typically bacterially produced in aquatic systems (Learman et al., 2011; Diaz et al., 2013). Dissolved organic matter, such as humic substances, can also reduce solid phase manganese oxides, resulting in the formation of Mn(III)-L (Oldham et al., 2017a).

Abiotic oxidation of Mn(II) by O_2_ is slow, but it can be accelerated by surface reactions (Morgan, 2000). However, bacteria are considered the major driving force behind manganese oxide formation in most aquatic environments (Tebo et al., 2004, 2005). From an evolutionary standpoint, the process of manganese oxidation is both phylogenetically and enzymatically diverse and the bacteria that carry out these processes are ubiquitous in nature. Due to the significance of these microorganisms in the geochemical cycling of manganese in aquatic and terrestrial systems alike, numerous studies have been undertaken to further understand the mechanisms surrounding oxide formation across different phyla. Many of these studies have demonstrated enzymes to be involved in the oxidation of Mn(II), including: MofA in *Leptothrix discophora* SS-1 (Corstjens et al., 1997), MoxA in *Pedomicrobium* sp. ACM 3067 (Ridge et al., 2007) and MopA in *Aurantimonas manganoxydans* SI85-9A1 (Anderson et al., 2009). While these are illustrative of single-enzyme mechanisms, many bacteria oxidize manganese through more complex systems or use multiple enzymes. For example, *Bacillus* sp. strains PL-12 and SG-1 employ a multi-subunit enzyme which includes the MnxG multicopper oxidase (van Waasbergen et al., 1996; Dick et al., 2008; Butterfield et al., 2013).

*Pseudomonas putida* GB-1 is a prominent model Mn-oxidizing bacterium that has multiple Mn oxidase genes: *mnxG*, which encodes a multicopper oxidase similar (albeit with low sequence similarity) to that found in *Bacillus* sp. PL-12 and SG-1 (Dick et al., 2008; Geszvain et al., 2013); *mcoA*, encoding a unique multicopper oxidase (Geszvain et al., 2013); and *mopA*, an animal heme peroxidase similar to *A. manganoxydans* SI85-9A1 MopA and regulated by the flagellar synthesis gene *fleQ* (Geszvain et al., 2016). Many pseudomonads, including *P. putida* GB-1 (as well as another well-characterized strain, *P. putida* MnB1), produce pyoverdines (PVD), a class of fluorescent siderophores that chelate Fe(III) for uptake in environments with low soluble iron concentrations. Investigations analyzing PVD produced by *P. putida* MnB1 revealed that PVD binds Mn(III) with a strength equal to or greater than that with which it binds Fe(III) and that when present during Mn(II) oxidation, PVD binds to Mn(III) produced as an intermediate in the oxidation of Mn(II) to Mn(IV) (Parker et al., 2014). Although studies have demonstrated that Mn(III) occurs as an enzymatic intermediate in Mn(II) → Mn(IV) oxidation and that siderophores can bind Mn(III) with high affinity, no studies have investigated the effect of the presence of complexing ligands with different binding strengths on the rate and extent of Mn oxide formation during growth and Mn(II) oxidation by Mn(II)-oxidizing bacteria. Additionally, it is unknown whether bacteria can oxidize Mn(III) bound to heterologous siderophores, i.e., a siderophores produced by other organisms.

In this work, we show that *Pseudomonas* species are able to directly oxidize Mn(III)-L to Mn(IV). As relatively high levels of Mn(III)-L have been detected in aquatic environments, it is likely that under certain conditions, Mn(III)-L accumulates and therefore, *Pseudomonas* and other Mn-oxidizing bacteria would affect the formation and direct oxidation of Mn(III)-L (Oldham et al., 2017b). Through these data, we show that *Pseudomonas* spp. can not only directly oxidize Mn(III)-L, but that when Mn(II) is oxidized in the presence of excess ligand, it first accumulates as a Mn(III)-L complex before undergoing a second oxidation step to Mn(IV). Given their ubiquity in nature, we propose that manganese-oxidizing members of the genus *Pseudomonas* potentially play a role in both the accumulation and consumption of Mn(III)-L complexes in aquatic systems, and ultimately in the overall geochemical cycling of manganese.

## Materials and methods

### Bacterial strains

#### *Pseudomonas hunanensis* GSL-007 and *Pseudomonas* sp. GSL-010

*Pseudomonas hunanensis* GSL-007 and *Pseudomonas* sp. GSL-010 (Table 1) were isolated from water samples obtained from Station 23, St. Lawrence Estuary (48°42.032″N, 68°39.171″W). Briefly, sampled waters were diluted 1:1,000 using sterile 0.9% NaCl and streaked onto Minimal Media A (MMA) plates enriched with 100 μM Mn(III)-citrate. Individual colonies that exhibited Mn(III,IV) oxide formation (dark brown colonies) were subsequently selected, grown in MMA medium and the formation of Mn(III,IV) oxide reconfirmed. Phylogeny was determined using 16S rRNA sequencing and analysis as previously described (Farooqui et al., 2016). The 16S rRNA GenBank accession numbers for *P. hunanensis* GSL-007 and *Pseudomonas* sp. GSL-010 are KY471136 and KY471137 respectively. *Pseudomonas* sp. GSL-010 is currently under consideration for publication as a novel species and is deposited in the Japan Collection of Microorganisms (ID = 32154), the Korean Collection for Type Cultures (ID = 62392) and the NITE Biological Research Center (ID = 113027).

**Table 1:** Bacterial strains and mutants used in this study.

**Strain or Mutant**	**Genotype, characteristics, or construction**	**References**
*Escherichia coli* TAM1	*mcrA* Δ(*mrr*-*hsdRMS*-*mcrBC*) ϕ80*lacZ*ΔM15 Δl*acX74 recA1 araD139* Δ(ara-leu)*7697 galU galK rpsL endA1 nupG*	Active Motif
*P. putida*^a^		
GB-1	Wild type (Amp^r^)^b^	Okazaki et al., 1997
KG_mnxGmopA	Δ*mcoA*	Geszvain et al., 2013
KG_TKO	Δ*mnxG* Δ*mcoA* Δ*mopA* Δ*fleQ*	Geszvain et al., 2016
KG_mcoA	Δ*mnxG* Δ*mopA*	This study
KG_mnxG	Δ*mcoA* Δ*mopA*	This study
KG_mopA	Δ*mnxG* Δ*mcoA* Δ*fleQ*	Geszvain et al., 2016
*P. hunanensis* GSL-007	Wild type	This study
*Pseudomonas* sp. GSL-010	Wild type	This study

a Pseudomonas putida GB-1 has three genes involved in Mn oxidation; mnxG, mcoA, and mopA.

b *Amp^r^, ampicillin resistant*.

#### *Pseudomonas* putida GB-1

*Pseudomonas putida* GB-1 was originally isolated by the research group of Kenneth Nealson from Green Bay, Wisconsin, United States of America (Okazaki et al., 1997). Mn oxidation mutants of *P. putida* GB-1 were those as previously described (Geszvain et al., 2013, 2016) or constructed prior to use in this study. Briefly, deletion construct plasmids pKG168 or pKG170 (Table 2) were moved into the relevant mutant strains via conjugation. Counter-selection against the *sacB* locus on the gene replacement vector was used to isolate double recombinants; deletion of the target gene was confirmed by PCR across the deletion site (Table 3).

**Table 2:** Plasmids used in this study.

**Plasmid**	**Description**	**Antibiotic Resistance**	**References**
pEX18Gm	Gene replacement vector, *oriT, sacB*	Gm^r^	Hoang et al., 1998
pRK2013	Helper plasmid for conjugation	Kn^r^	Ditta et al., 1980
pKG170	*mcoA* deletion construct	Gm^r^	Geszvain et al., 2013
pKG168	*mopA* deletion construct	Gm^r^	Geszvain et al., 2016

*Gm^r^, gentamicin resistant; Kn^r^, kanamycin resistant*.

**Table 3:** Primers used in this study.

**Primer name**	**Purpose**	**Sequence**
2665_upstream-F	Amplify across *mcoA* deletion	5′ CCAGGTCGGCTCGTTCTGGCG 3′
2665_downstream-R	Amplify across *mcoA* deletion	5′ AGGCCATCGATCCACAGCCCCAG 3′
Per-upstream-F	Amplify across *mopA* deletion	5′ CCTCCCTTTATCGCTAAGCGGG 3′
Per-downstream-R	Amplify across *mopA* deletion	5′ AGAAGAACCGCCTGGTGGC 3′

### Culture conditions

Strains used in this study are strictly aerobic mesophiles and were maintained in (Luria-Bertani Broth) LB medium, comprised of (per liter of diH_2_O): 10 g Bacto™ Tryptone (Becton, Dickinson and Company; MD, USA), 5 g yeast extract (Becton, Dickinson and Company; MD, USA) and 10 g NaCl with the pH adjusted to 7.5 using 1 M HCl/NaOH prior to autoclaving. For all quantitative analyses, Minimal Media A (MMA) was utilized and comprised of (per liter of Milli-Q H_2_O): 0.238 g (NH_4_)_2_SO_4_, 0.0602 g MgSO_4_, 0.0488 g CaCl_2_.2H_2_O, 0.0204 g KH_2_PO_4_ and 0.0284 g Na_2_HPO_4_ before autoclaving, with sterile 4.5 mL 20% glucose, 200 μL 5x Trace Elements Mix (Atlas, 2010), 1 mL 3.7 mM FeCl_3_.6H_2_O and 20 mL 1 M HEPES (pH 7.8) added after autoclaving with 100 μM Mn(II) (as MnCl_2_), Mn(III)-citrate, or Mn(III)-DFOB added as required. Autoclaving was completed under standard conditions (121°C for 15 min under 1.05 kg/cm^2^). All cultures were grown and maintained at 30°C and experiments were performed in triplicate. All glassware and other apparatus were acid washed prior to use.

### Synthesis of Mn(III)-L compounds

#### Mn(III)-citrate

Mn(III)-citrate was used as a model weak Mn(III)-L complex. Stock solutions were prepared through the dissolution of Mn(III)-acetate in the presence of excess sodium citrate. Initially, 30 mM of sodium citrate (Fisher Scientific) was added to Milli-Q water and adjusted to pH 7 using trace metal clean HCl/NaOH. Ten millimolar Mn(III)-acetate was added, vigorously shaken, readjusted to pH 7 and allowed to sit in the absence of light for 24 h. Following this, the solution was 0.22 μm sterile filtered to remove any produced Mn(III,IV) oxide. Concentration of Mn(III)-citrate was determined spectrophotometrically at A_428_; ε = 310 L·mol^−1^·cm^−1^ (Duke, 1947).

#### Mn(III)-desferrioxamine B [Mn(III)-DFOB]

Mn(III)-DFOB was used as a model strong Mn(III)-L complex. Stock solutions were prepared through air oxidation of Mn(II) in the presence of DFOB mesylate salt (Sigma Aldrich) using a method adapted from Duckworth and Sposito (2005). Initially, 2 mM DFOB mesylate salt was added to Milli-Q water and adjusted to pH 9 using trace metal clean HCl/NaOH. 1.1 mM MnCl_2_ was then added and the solution oxygenated for 24 h using filtered air. The solution was then readjusted to pH 8 and 0.22 μM sterile filtered to remove any produced Mn(III,IV) oxide. Final Mn(III)-DFOB concentration was determined spectrophotometrically at A_310_; ε = 2055 L·mol^−1^·cm^−1^. Mn(III)-DFOB recovery was ≥97% and produced Mn(III)-DFOB complex is stable through pH 7–11.3 and was used within 30 days of synthesis.

### Chemical analyses

#### Leucoberbelin blue (LBB) assay

Quantification of formed Mn(III)-L_(weak)_ and Mn(III,IV) oxide was performed colorimetrically using an adapted LBB assay (Krumbein and Altman, 1973; Tebo et al., 2007). Stock solutions of 4% LBB were prepared using Milli-Q H_2_O, 24 mM NaOH and stored in the absence of light at 4°C. For use in assaying, LBB stock was diluted 1:100 in 1% glacial acetic acid (v/v) (storage life ~1 week). Standard curves of 10–100 μM were generated using known concentrations of fresh (≤6 h) KMnO_4_. Samples were visualized spectrophotometrically at A_623_ using a SpectraMax M2^e^ (Molecular Devices LLC; CA, USA). LBB measures the oxidizing equivalents of Mn with an oxidation state > +II. Thus, one mole of Mn(VII) (KMnO_4_) will oxidize 5 moles of LBB. When calculating Mn(III,IV) oxide concentrations, it was assumed that all particulate Mn was MnO_2_.

### Growth consistency and enumeration of cells

For Mn(III)-L oxidation and production experiments, growth rates were synchronized to ensure consistency of results. Initially, frozen glycerol culture stocks (−80°C, 2:1 80% glycerol/log phase culture) were streaked onto LB agar plates. Individual colonies were then streaked to MMA agar plates and transferred to MMA after 24 h. For consistency of results, throughout all analytical experiments relative growth between replicates and between strains was monitored spectrophotometrically at OD_600_ (data not shown) to ensure the replicates for each given strain grew similarly. All experiments were performed in the absence of light unless otherwise specified.

### Mn(III)-L oxidation experiments

For Mn(III,IV) oxide formation experiments, the *Pseudomonas* strains were grown in 5 mL aliquots of MMA media-containing 100 μM of either Mn(II), Mn(III)-citrate or Mn(III)-DFOB. Cultures were vacuum filtered through a 0.45 μm filter disk (Durapore), both the tube and disk were washed with 5 mL MilliQ H_2_O and the filter disk was returned to the original tube for analysis. Mn-oxides were observed on filter disks and were colorimetrically determined using 0.04% LBB. Sampling occurred at h 0, 8, 16, 24, 48, 96 for Mn(II)/Mn(III)-citrate and h 0, 24, 48, 72, 120, 168 for Mn(III)-DFOB. The maximum rate of Mn(III,IV) oxide formation was determined from the line between the two points that showed the steepest slope.

### Mn(III)-L formation experiments

Our experimental design for Mn(III)-L formation experiments was formulated to both prevent iron limitation and siderophore production by the *Pseudomonas* isolates, as well as to avoid abiotic Mn(III)-DFOB production before biotic oxidation could proceed. To achieve this, the *Pseudomonas* strains or mutants were grown for 16 h in 300 mL of MMA media and monitored to ensure no siderophores were produced (fluorescence of pyoverdines). The growth consistency was confirmed and then each culture was amended with 100 μM Mn(II), as well as 2 mM Na-citrate, or 150 μM DFOB mesylate salt. These cultures were then dispensed into 5 mL aliquots. At various times, cultures were vacuum filtered through a 0.45 μm filter disk (Durapore), both tube and disk washed with 5 mL Milli-Q H_2_O and the filter disk was returned to the original tube for analysis as described above. Mn-oxides were observed on filter disks and were colorimetrically quantified using 0.04% LBB. Mn(III)-citrate present in the filtrate was determined using 0.04% LBB. Mn(III)-DFOB present in the filtrate was determined spectrophotometrically at A_310_; ε = 2,055 L·mol^−1^·cm^−1^. Sampling occurred at h 0, 8, 16, 24, 48, 96 for Mn(II)/Mn(III)-citrate and h 0, 24, 48, 72, 120, 168 for Mn(III)-DFOB. Unlike removal experiments, the Mn source and ligand were added after growth reached log phase to avoid chelation with free iron in the medium (see section Discussion).

## Results

### Mn(III)-L oxidation by St. Lawrence estuary isolates

**Figure 1:**
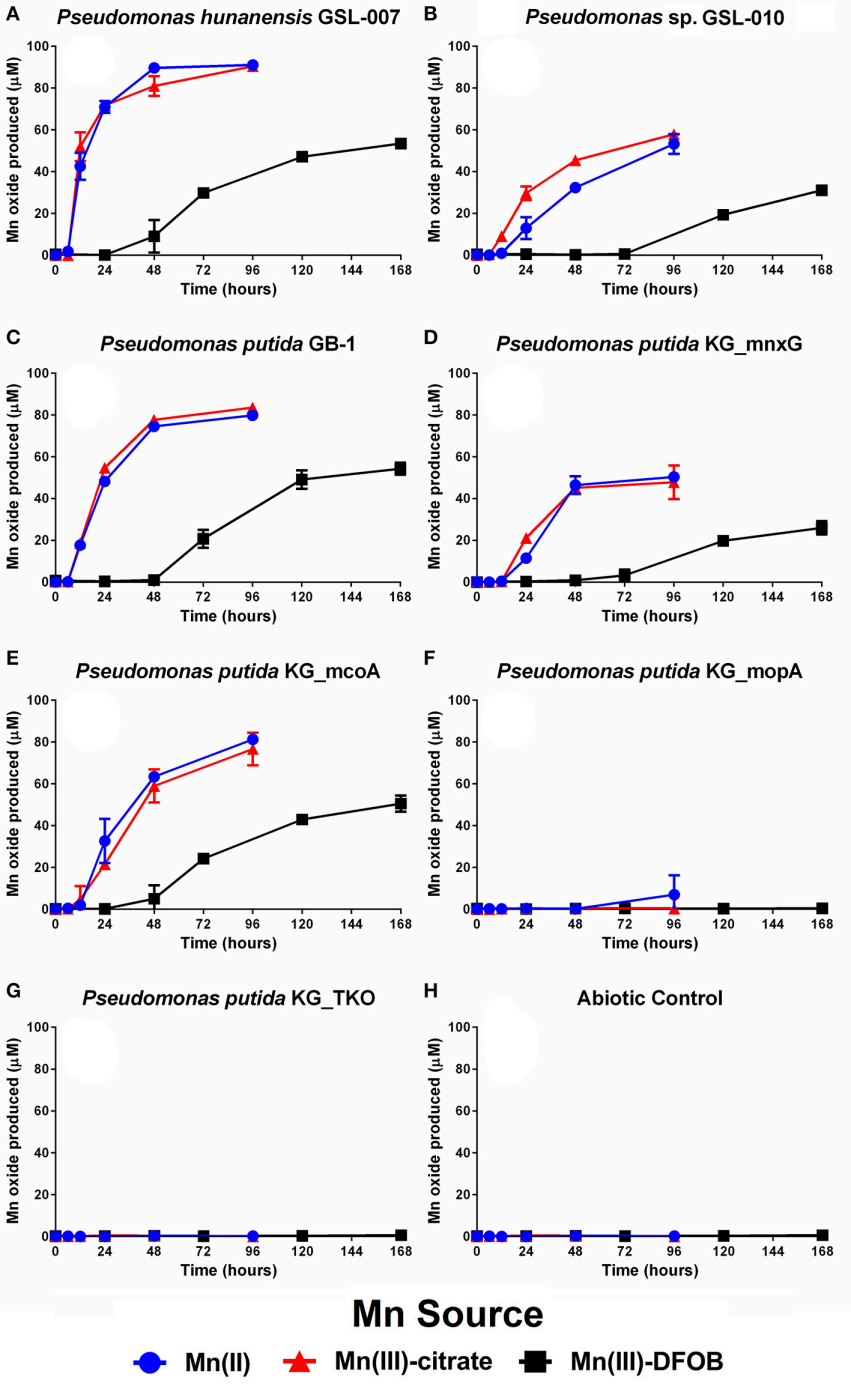
Mn(II), Mn(III)-citrate, and Mn(III)-DFOB oxidation over time by **(A)** *P. hunanensis* GSL-007, **(B)** *Pseudomonas* sp. GSL-010, **(C)** *P. putida* GB-1 alongside associated mutants—**(D)** *P. putida* KG_mnxG (producing only MnxG), **(E)** *P. putida* KG_mcoA (producing only McoA) and **(F)** *P. putida* KG_mopA (producing only MopA)—as measured by Mn(III,IV) oxide formation. Mn(II) was used as a positive control with **(G)** sterile medium and **(H)** *P. putida* TKO (devoid of any Mn oxidase genes) serving as negative controls. Grown in minimal media at 30°C containing 100 μM of relevant Mn compound. Relative growth between bacterial isolates was normalized by OD_600_ (results not shown).

The ability of *P. hunanensis* GSL-007 (Figure 1A) and *Pseudomonas* sp. GSL-010 (Figure 1B) to directly oxidize Mn(III)-L over time was assessed using Mn(III)-citrate (weakly complexed ligand), Mn(III)-DFOB (strongly complexed ligand), with Mn(II) as a positive control. *P. putida* TKO (Figure 1G) was used as a negative control and did not exhibit any Mn oxidation. Both environmental isolates were able to oxidize all manganese species tested, with the rate of production and final yield from all three being higher for *P. hunanensis* GSL-007 than *Pseudomonas* sp. GSL-010 (Figures 1A,B; quantitative data shown in Table 4). Mn(II) and Mn(III)-citrate oxidation were oxidized at the same rate, with Mn(III,IV) oxide initially detected after 8–16 h and maximum oxidation detected after 48–96 h. However, oxidation of Mn(III)-DFOB was significantly slower, with initial oxidation evident after 48–120 h and maximum oxidation observed after 168 h. There were also significant differences observed in the final quantity of Mn(III,IV) oxide formed depending on the initial substrate, with Mn(II) and Mn(III)-citrate producing equal amounts of Mn(III,IV) oxide and Mn(III)-DFOB producing significantly less (Table 4).

**Table 4:** Final yields of Mn(III,IV) oxide formed through bacterial Mn(II/III) oxidation (see Figure 1) and maximum rates.

	**Mn(II)**		**Mn(III)-citrate**		**Mn(III)-DFOB**	
**Strain or mutant**	**Mn(III,IV) oxide (μM)**	**Maximum rate (μM/h)**	**Mn(III,IV) oxide (μM)**	**Maximum rate (μM/h)**	**Mn(III,IV) oxide (μM)**	**Maximum rate (μM/h)**
*P. putida* GB-1	79.8 ± 2.0	2.9	83.5 ± 0.6	3.1	54.3 ± 2.9	0.8
*P. putida* KG_mcoA	81.3 ± 1.5	2.6	76.7 ± 7.8	1.5	50.6 ± 0.9	0.8
*P. putida* KG_mnxG	50.4 ± 1.3	1.5	47.8 ± 8.0	1.7	26.0 ± 3.2	0.3
*P. putida* KG_mopA	7.0 ± 9.2	0.1	0.0 ± 0.0	0.0	0.1 ± 0.0	0.0
*P. hunanensis* GSL-007	91.1 ± 1.9	6.7	90.4 ± 1.3	8.6	53.5 ± 0.8	0.9
*Pseudomonas* sp. GSL-010	53.2 ± 4.7	1.0	57.9 ± 1.5	1.5	31.1 ± 1.1	0.4

*Initial Mn source was added as 100 μM. Formed Mn(III,IV) oxide values presented are from the final timepoint from each culture: 96 h in Mn(II)/Mn(III)-citrate cultures and 168 h in Mn(III)-DFOB cultures*.

### Mn(III)-L oxidation between *Pseudomonas putida* GB-1 and Mn oxidase mutants

The specificity of *P. putida* enzymes MnxG, McoA, and MopA toward Mn(III) was tested using the wildtype strain GB-1, alongside mutants *P. putida* KG_mnxG (only produces MnxG), *P. putida* KG_mcoA (only produces McoA) and *P. putida* KG_mopA (only produces MopA) (Figures 1C–F; Table 1). Mn(II) and Mn(III) oxidation were observed with *P*. *putida* GB-1, *P. putida* KG_mnxG and *P. putida* KG_mcoA; however, P. *putida* KG_mopA failed to oxidize Mn(III) and showed only limited Mn(II) oxidation after 96 h (Figure 1F; Table 4). Because *P. putida* KG_mopA showed little to no activity under these conditions, the remainder of the results describe just the wildtype, *P. putida* KG_mnxG and *P. putida* KG_mcoA strains. As was seen with the St. Lawrence estuary isolates, the *P. putida* GB-1 strains oxidized Mn(II) and Mn(III)-citrate at similar rates, while oxidation of Mn(III)-DFOB was much slower (Figures 1C–E; Table 4). For each of the three tested Mn compounds, the strain *P. putida* KG_mnxG produced lower quantities of Mn(III,IV) oxide than wildtype, *P. putida* KG_mcoA and *P. hunanensis* GSL-007 but roughly similar amounts as *Pseudomonas* sp. GSL-010 (Table 4).

#### Mn(III)-citrate accumulation

**Figure 2:**
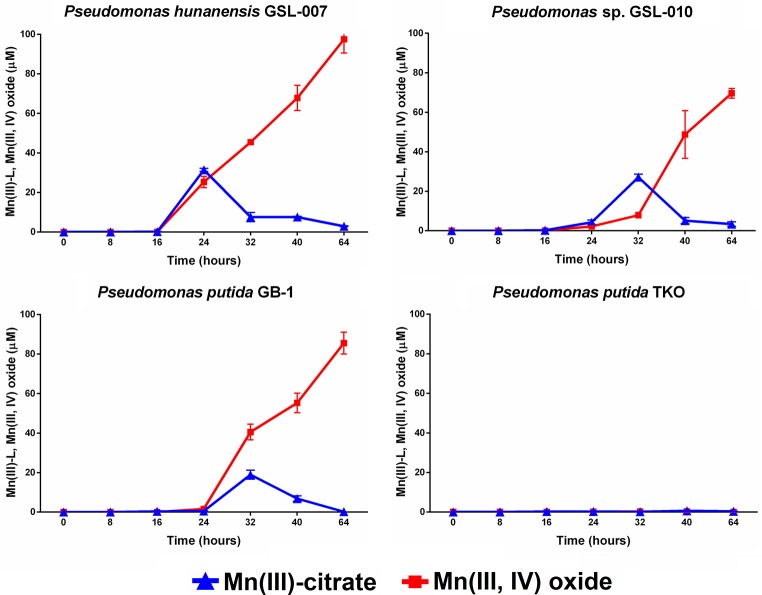
Mn(III)-citrate and Mn(III,IV) oxide accumulation during Mn(II) oxidation from *Pseudomonas putida* GB-1, *P. hunanensis* GSL-007 and *Pseudomonas* sp. GSL-010. *P. putida* TKO served as a negative control. Cultures were grown for 16 h in MMA at 30°C before addition of 100 μM MnCl_2_ and 2 mM sodium citrate. Relative growth between bacterial isolates was normalized by OD_600_ (results not shown).

Mn(II) oxidation by *P. putida* GB-1, *P. hunanensis* GSL-007 and *Pseudomonas* sp. GSL-010, in the presence of excess sodium citrate, was tested to detect accumulation of intermediary, weakly-bound Mn(III)-L (Figure 2; Table 5). There was no Mn(III,IV) formation observed in the triple knockout mutant (*P. putida* TKO). Mn(III)-citrate formation was detected at 8–16 h in all oxidizing cultures and remained at low concentrations during Mn(III,IV) oxide formation; however, the Mn(III)/Mn(IV) ratios observed were different for the tested isolates. For *P. putida* GB-1, Mn(III,IV) oxide was the primary product detected (40.6 ± 4.0 μM) after 16 h, compared to Mn(III)-citrate (19.0 ± 3.8 μM). The opposite trend was observed in *Pseudomonas* sp. GSL-010, with Mn(III)-citrate (27.1 ± 1.5 μM) present at greater concentrations than Mn(III,IV) oxide (7.9 ± 1.4 μM). Relatively similar levels of Mn(III)-citrate (31.5 ± 0.8 μM) and Mn(III,IV) oxide (45.5 ± 0.3 μM) were detected in *P. hunanensis* GSL-007 at initial formation after 8 h. After 48 h, Mn(III,IV) oxide formation was observed (69.6–85.6 μM) under all conditions and almost no Mn(III)-citrate (0.2–3.4 μM) was detected.

**Table 5:** Maximum Mn(III)-citrate observed along with final yields of Mn(III)-citrate and Mn(III,IV) oxide formed through bacterial Mn(II) oxidation (see Figure 2).

**Strain**	**Maximum Mn(III)-citrate (μM)**	**Mn(III)-citrate (Final)**	**Final Mn(III,IV) oxide concentration (μM)**
*P. putida* GB-1	18.9 ± 2.7 (at 32 h)	0.2 ± 0.0	85.5 ± 5.5
*P. hunanensis* GSL-007	31.5 ± 0.8 (at 24 h)	2.9 ± 0.2	97.4 ± 6.9
*Pseudomonas* sp. GSL-010	27.1 ± 1.5 (at 32 h)	3.4 ± 1.1	69.6 ± 2.5

*Final yields of Mn(III)-citrate and Mn(III,IV) oxide values presented are from 64 h*.

#### Mn(III)-DFOB accumulation

**Figure 3:**
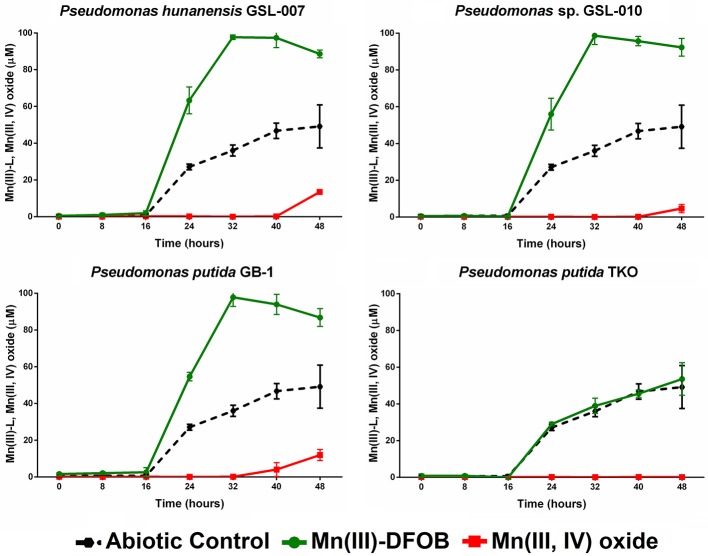
Mn(III)-DFOB and Mn(III,IV) oxide accumulation during Mn(II) oxidation from *Pseudomonas putida* GB-1, *P. hunanensis* GSL-007 and *Pseudomonas* sp. GSL-010. *P. putida* TKO was used as a negative control and abiotic oxidation was measured over time in culture-free medium. Cultures were grown for 16 h in MMA at 30°C before being inoculated with 100 μM MnCl_2_ and 150 μM DFOB. Relative growth between bacterial isolates was normalized by OD_600_ (results not shown).

Mn(II) oxidation by *P. putida* GB-1, *P. hunanensis* GSL-007 and *Pseudomonas* sp. GSL-010 in the presence of excess DFOB was tested to assess accumulation of intermediary, strongly-bound Mn(III)-L (Figure 3; Table 6). Substantial accumulation of Mn(III)-DFOB was observed for both the abiotic and *P. putida* TKO negative controls. DFOB is known to promote the oxidation of Mn(II) by oxygen resulting in the formation of Mn(III)-DFOB (see section Materials and Methods; Duckworth and Sposito, 2005) However, bacterial oxidation proceeded at greater rates than the abiotic control, with all isolates showing complete (≥97.5 μM) Mn(III)-DFOB formation after 32 h. Comparatively, at the same timepoint abiotic Mn(III)-DFOB formation was observed at levels of 36.0 ± 3.0 μM. Similar trends were observed between all bacterial isolates, with initial Mn(III)-DFOB formation observed followed by Mn(III,IV) oxide formation 40–48 h after initial inoculation. Mn(III,IV) oxide formation was not observed in the abiotic control but was observed in all oxidizing cultures (4.7–13.4 μM) and occurred as Mn(III)-DFOB concentrations decreased, indicative of a secondary oxidation step. There was no significant increase in Mn(III)-DFOB formation in the triple knockout mutant (*P. putida* TKO) relative to the abiotic control.

**Table 6:** Final yields of Mn(III)-DFOB and Mn(III,IV) oxide formed through bacterial Mn(II) oxidation (see Figure 3).

**Strain**	**Mn(III)-DFOB (μM)**	**Mn(III,IV) oxide (μM)**
*P. putida* GB-1	86.8 ± 4.9	12.0 ± 3.0
*P. hunanensis* GSL-007	88.6 ± 2.1	13.4 ± 0.5
*Pseudomonas* sp. GSL-010	92.3 ± 4.8	4.7 ± 2.2
Abiotic control	49.2 ± 11.7	0.1 ± 0.1

*Final yields of Mn(III)-DFOB and Mn(III,IV) oxide values presented are from 48 h*.

## Discussion

Research into bacterial Mn(II)-oxidizing enzymes has highlighted that these oxidases produce Mn(III) as an intermediate during the formation of particulate Mn(III,IV) oxide and are also capable of directly oxidizing Mn(III). The multicopper oxidase (MCO) in *Bacillus* sp. PL-12 (containing a MnxG subunit with low levels of homology to *P. putida* GB-1) catalyzes two single one-electron oxidation steps, from Mn(II) to Mn(III) and then Mn(III) to Mn(IV) (Soldatova et al., 2017a,b). Similarly, animal heme peroxidases produced by *A. manganoxydans* SI85-9A1, *Erythrobacter* sp. SD-21 and *Roseobacter* sp. Azwk-3b oxidized Mn(II) to Mn(III), which subsequently disproportionated or was further oxidized to Mn(IV) (Anderson et al., 2009; Andeer et al., 2015). Though illustrative of Mn(III) oxidation/complexation, these studies used purified protein or the exosporium, whereas this study examines these processes when carried out by bacterial cultures. Given the frequent occurrence of *Pseudomonas* in different environments, members of the genus were selected as model organisms to further understand the bacterial influence on Mn(III)-L formation and removal in aquatic systems. This included species isolated from the St. Lawrence Estuary (*P. hunanensis* GSL-007 and *Pseudomonas* sp. GSL-010) as well as *P. putida* GB-1, an Mn(II)-oxidizer long studied as a laboratory model. *P. putida* GB-1 encodes two multicopper oxidases (MnxG and McoA) and an animal heme peroxidase (MopA) implicated in Mn oxidation (Geszvain et al., 2013, 2016). Mn(III) oxidation in *P. putida* GB-1 was examined with mutants expressing only one of these genes in order to determine whether a specific oxidase, or a group of enzymes, interacts with Mn(III).

DFOB, a siderophore produced by some bacteria and fungi, was selected as a model strong complexing ligand because siderophores are produced by a wide range of bacteria and fungi under low iron stress in environments (such as aquatic systems) where iron is not always readily available and are environmentally significant (Neilands, 1995). Indeed, both *P. putida* GB-1 and the closely related strain, *P. putida* MnB1, produce siderophores under iron-starved conditions; these siderophores were found to have a high binding affinity for Mn(III) and inhibit Mn(IV) formation (Parker et al., 2004, 2014). Given that siderophores (including DFOB and the *P. putida*-produced siderophores) have a natural affinity to bind to iron, one consideration in our experimental design was the necessity to avoid chelation between the DFOB and free iron in the culture media during growth phase. A second consideration was that abiotic Mn(III)-DFOB synthesis can occur through the air oxidation of Mn(II) in the presence of DFOB (Figure 3; Duckworth and Sposito, 2005). We saw no evidence of siderophore production (fluorescence of pyoverdines known to be produced by *P. putida* GB-1) during the growth phase of our incubations and although abiotic oxidation of Mn(II) was observed in the DFOB experiments, Mn-oxidizing bacteria formed Mn(III)-DFOB at a faster rate. The low levels of Mn(III,IV) oxide production observed after 48 h relative to the Mn(II) and Mn(III)–citrate experiments is likely due to the reduction of Mn(III,IV) oxide by DFOB (Oldham et al., 2015) resulting in an apparent decrease in the rate of formation due to a decrease in Mn(III,IV) oxide accumulation.

Direct Mn(III)-L oxidation was observed in wild type cultures of *P. hunanensis* GSL-007, *Pseudomonas* sp. GSL-010, and *P. putida* GB-1 as well as by mutants *P. putida* KG_mcoA and *P. putida* KG_mnxG (Figure 1). Conversely, no Mn(III)-L oxidation was detected in *P. putida* KG_mopA cultures and very little particulate Mn(III,IV) oxide formation was observed in the Mn(II) control. These results contrast previous investigations of MopA, which after 72 h showed significant Mn(III,IV) oxide formation (Geszvain et al., 2016). This difference can be explained by the effect of light. Our experiments were performed in the dark, while those reported by Geszvain et al. (2016) were exposed to ambient room light. A separate experiment with *P. putida* KG_mopA cell-free extracts showed that MopA produces very little Mn(III,IV) oxide in the dark compared to relatively high levels in the light (Data Sheet 1). A similar effect of light has been demonstrated in *Roseobacter* sp. AzwK-3b, another species that utilizes a MopA homolog in Mn oxidation (Hansel and Francis, 2006). However, by conducting our experiments in the dark, we intended to better replicate the environmental conditions from which our isolates were obtained and to avoid any potential photoreactions with the HEPES or photoreduction of Mn in our growth media.

One longstanding question surrounding *P. putida* GB-1 is the rationale behind the presence of multiple Mn oxidase enzymes, with one hypothesis being that they are activated under different environmental conditions (Geszvain et al., 2016). Given the dependence on light, MopA may be expressed predominately in the upper regions of the oxic zone of aquatic environments where light is abundant, whereas in the lower regions where light is absent, it may not be expressed. However, this hypothesis assumes that MopA-driven oxidation is beneficial in the euphotic zone and not at deeper levels. Even though the Mn(II)-oxidizing peroxidases from both *P. putida* GB-1 and *Roseobacter* sp. AzwK-3b are thought to perform single, one electron oxidation reactions [Mn(II) to Mn(III)], both appear to produce Mn(III,IV) oxide. Similarly, Mn(III/IV) oxide formation was observed in our MopA light/dark experiments through the enzymatic oxidation of Mn(II). How peroxidases catalyze formation of solid Mn(III,IV) oxide requires further investigation.

Citrate was selected as a model weak complexing ligand because *P. hunanensis* GSL-007 and *Pseudomonas* sp. GSL-010 were originally isolated by their ability to form Mn(III,IV) oxide from Mn(III)-citrate. We observed that in the presence of excess citrate, oxidation of Mn(II) would lead to complexation of Mn(III) to form Mn(III)-citrate before a later secondary oxidation step to form Mn(III,IV) oxide. Though our results highlight the first instance of direct bacterial oxidation causing Mn(III)-L accumulation, trapping of an Mn(III)-intermediate using the ligand pyrophosphate (PP) and subsequent oxidation to Mn(IV) has been demonstrated using exosporium containing the intact Mnx complex of *Bacillus* sp. SG-1 (Webb et al., 2005; Soldatova et al., 2012) and the purified Mnx complex from *Bacillus* sp. PL-12 (Soldatova et al., 2017a,b). Given the weak complexation of Mn(III) to PP, we predicted that Mn(III)-citrate would behave in a similar manner. Because the HEPES we used in our media can abiotically reduce Mn(III)-pyrophosphate (Kostka and Luther, 1994), we used Mn(III)-citrate as the model weak complexing ligand. When we substituted a phosphate buffer for HEPES and tested for pyrophosphate, all isolates were capable of Mn(III,IV) oxide formation using Mn(III)-pyrophosphate (results not shown). There was no observed abiotic reduction of Mn(III)-citrate by HEPES under the experimental conditions utilized in this study.

While our findings prompt a reevaluation of the process of bacterial Mn(II, III) oxidation in aquatic environments, it is important to note that recent investigations into reductive processes have also fundamentally changed our understanding of these systems. Recently, it was learned that Mn(IV) reduction requires an initial solubilization step and that Mn(IV) reduction is in fact a series of two consecutive one-electron transfer reactions, with soluble Mn(III) produced as intermediary species (Lin et al., 2012). Subsequent investigations of a bacterial isolate, *Shewanella oneidensis*, highlighted the process of direct Mn(III) reduction and provided insight into the associated mechanisms (Szeinbaum et al., 2014). Given that reduction also involves formation of an Mn(III) intermediate, complexation to surrounding ligands is likely to occur as is observed in manganese oxidation. Previous work from members of our group, Oldham et al. (2015, 2017a), highlighted that one of the potential Mn(III)-L formation pathways is through the bacterial reduction of Mn(IV) in the presence of suitable ligands, resulting in complexation and formation of Mn(III)-L. Thus, while our results highlight the potential significance of Mn oxidizers in the formation and removal of Mn(III) in aquatic systems, any reassessment of the current cycling model should also incorporate Mn-reducing bacteria.

From an aquatic sciences standpoint, traditional research has focused almost exclusively on the reduction of Mn(IV) and the oxidation of Mn(II) based on operationally defined soluble, reduced Mn(II) (which passes through a 0.2 μm or 0.45 μm filter) and oxidized particulate Mn(III,IV) oxide trapped on the filters. However, an increased understanding into the significance of Mn(III) has prompted a paradigm shift in how different manganese species are cycled within environmental systems (Trouwborst et al., 2006; Madison et al., 2011; Luther et al., 2015; Oldham et al., 2017a,b). Though it has previously been suggested that bacterial oxidative processes likely drive the presence of Mn(III)-L in aquatic systems (Webb et al., 2005), this paper shows the first instance of direct bacterial Mn(III)-L formation and removal. Evidence of Mn(III)-L oxidation by MnxG and McoA, but not MopA suggests that this step may be predominately MCO driven, at least in the absence of light. While this research focused solely on *Pseudomonas* spp., given the ubiquity of manganese-oxidizing bacteria in aquatic environments and the influence they have on Mn(II), *Pseudomonas* and many other genera are also likely some of the driving forces behind Mn(III)-L formation and removal in the environment. Investigating whether these findings apply to other Mn(II) oxidizers may provide further insight into their influence on the cycling of Mn(III) in aquatic systems.

## Author contributions

The concepts being tested in this manuscript resulted from the collaboration between MW, BT, VO, and GL on Mn(III) cycling in the environment. MW and BT designed and performed the Mn oxidation experiments. KG generated the mutants and completed the light/dark experiments. VO and GL provided guidance on the Mn(III) analyses. All authors participated in the interpretation of the results and preparation of the manuscript.

### Conflict of interest statement

The authors declare that the research was conducted in the absence of any commercial or financial relationships that could be construed as a potential conflict of interest.
