# Supplementary material for: Oxidative Formation and Removal of Complexed Mn(III) by Pseudomonas Species
Source: Front Microbiol. 2018 Apr 12;9:560. doi: 10.3389/fmicb.2018.00560 (PMC5906577; doi:10.3389/fmicb.2018.00560)
Supplement: Supplementary file 1 [file DataSheet1.DOCX]

**SUPPLEMENTARY MATERIAL**

**MATERIALS AND METHODS**

**Preparation of cell-free culture supernatants:**

Cell-free culture supernatants were generated from strains *P. putida* KG_mopA and *P. putida* KG_TKO as in **Geszvain et al. 2016**. The cultures for generating these supernatants were grown 48 h at room temperature in the presence or absence of light.

**Cell-free culture experiments:**

To assay Mn(II) oxidation activity in light versus dark conditions, MnCl_2_ was added to the supernatants to a final concentration of 100 µM. 87.5 µL of the supernatant + Mn(II) mixture and 12.5 µL 10 mM HEPES pH 7.5 buffer were then added to individual wells of two identical 96 well plates. As a negative control, 87.5 µL supernatant with no MnCl_2_ and 12.5 µL 10 mM HEPES pH 7.5 were added to the same 96 well plates. Three identical reactions were set up for each supernatant, plus and minus MnCl_2_, for a total of 6 wells for each strain on each plate. Both plates were wrapped in plastic wrap but only one – designated the dark plate – was also wrapped in foil. They were placed together on a shelf under a fluorescent light that remained on continuously. After ~16 hr, 25 µL 0.04 % LBB was added to each well and absorbance at 618 and 700 nm recorded. For background correction, the absorbance at 700 nm was subtracted from the absorbance at 618 nm. The values for the negative control were subtracted from that of the supernatant and MnCl_2_ samples to estimate relative levels of oxides produced in the presence and absence of light.

**RESULTS**

**Cell-free culture experiments**

As has been previously observed during growth in culture (**Geszvain et al. 2016**), Mn(II) oxidation by MopA appears to be stimulated by light. To quantify this effect, we isolated and concentrated cell-free culture supernatant from 2 day old cultures of *P. putida* KG_mopA and *P. putida* KG_TKO, and estimated the yield of Mn(III,IV) oxide produced after incubation in light vs dark conditions. The *P. putida* KG_mopA cell-free culture supernatant produces nearly 10-fold more Mn(III,IV) oxide in the light than in the dark. There was no oxide production by the *P. putida* KG_TKO supernatant in either condition. While the amount of oxide production varies between preparations of cell-free culture supernatant, the effect of light remains consistent (data not shown).

**SUPPLEMENTARY FIGURES**


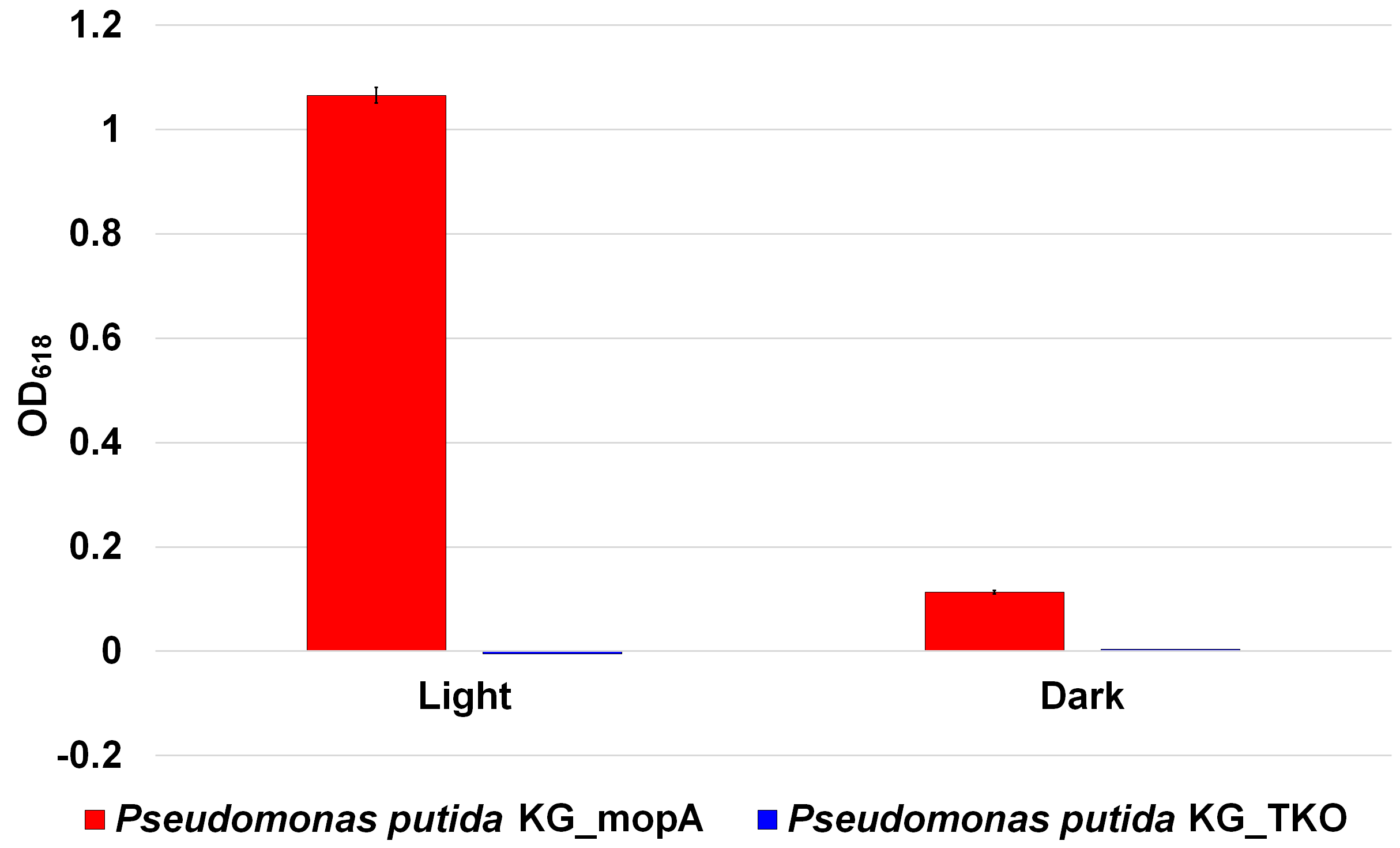


**Supplementary Figure 1:** Mn(II) oxidation by cell-free culture supernatants of *P. putida* KG_mopA and *P. putida* KG_TKO. Supernatants were inoculated with 100 µM MnCl_2_ and incubated for ~16 h in the presence or absence of light. LBB was then added and measured at OD_618_ to quantify Mn(III,IV) oxide formation. Experiment was performed in triplicate; errors bars represent standard deviation.
